# Supplementary figures and images for: Combating the Dust Devil: Utilizing Naturally Occurring Soil Microbes in Arizona to Inhibit the Growth of Coccidioides spp., the Causative Agent of Valley Fever
Source: J Fungi (Basel). 2023 Mar 11;9(3):345. doi: 10.3390/jof9030345 (PMC10056400; doi:10.3390/jof9030345)

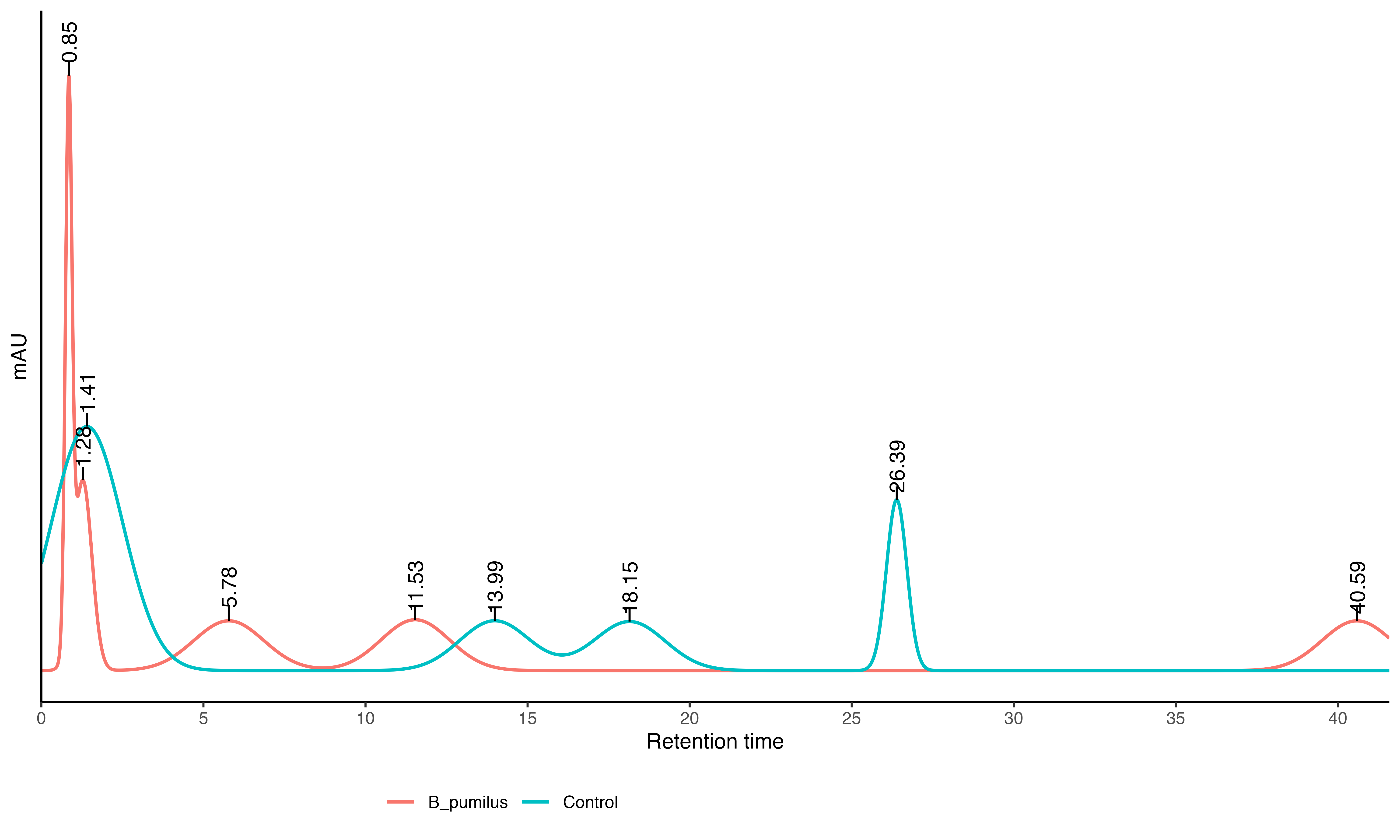

Supplement: Supplementary file 1 [file jof-09-00345-s001.zip › Figure S1.tiff]

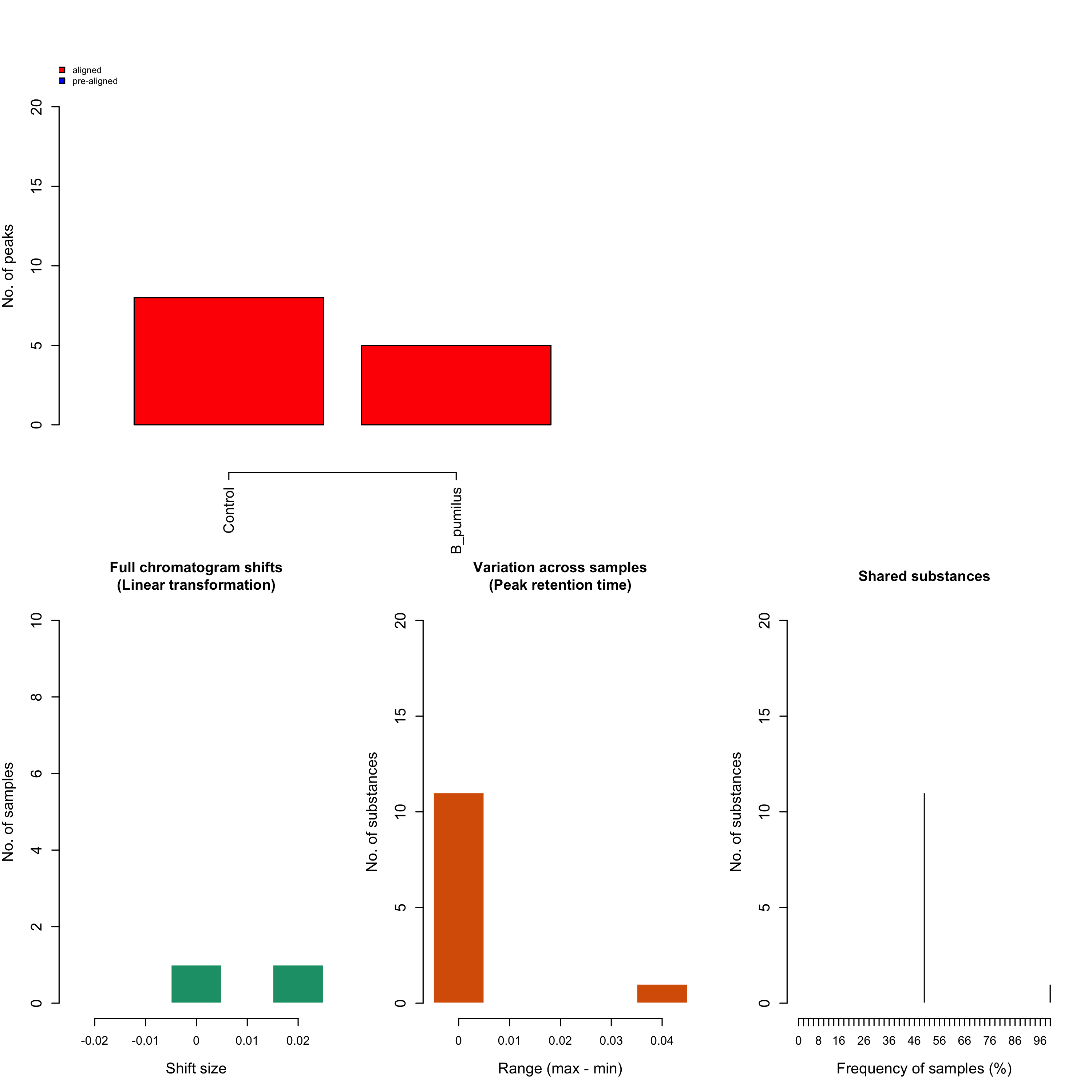

Supplement: Supplementary file 1 [file jof-09-00345-s001.zip › Figure S2.tiff]
